# *Lactobacillus rhamnosus GG* ameliorates radiation-induced lung fibrosis via lncRNASNHG17/PTBP1/NICD axis modulation

**DOI:** 10.1186/s13062-023-00357-x

**Published:** 2023-01-12

**Authors:** Zhao Ju, Huiji Pan, Can Qu, Liang Xiao, Meiling Zhou, Yin Wang, Jinhua Luo, Liangfang Shen, Pingkun Zhou, Ruixue Huang

**Affiliations:** 1 Department of Occupational and Environmental Health, Xiangya School of Public Health, Central South University, Changsha, 410078 Hunan Province China; 2 Department of Radiation Biology, Beijing Key Laboratory for Radiobiology, Beijing Institute of Radiation Medicine, AMMS, Beijing, China; 3 Faculty of Naval Medicine, Naval Medical University (Second Military Medical University), Shanghai, 200433 China; 4 Department of Oncology, Xiangya Hospital, Central South University, Changsha, China

**Keywords:** Radiation-induced pulmonary fibrosis, *Lactobacillus rhamnosus GG*, lncRNA, Epithelial–mesenchymal transition

## Abstract

Radiation-induced pulmonary fibrosis (RIPF) is a major side effect experienced for patients with thoracic cancers after radiotherapy. RIPF is poor prognosis and limited therapeutic options available in clinic. *Lactobacillus rhamnosus GG* (LGG) is advantaged and widely used for health promotion. However. Whether LGG is applicable for prevention of RIPF and relative underlying mechanism is poorly understood. Here, we reported a unique comprehensive analysis of the impact of LGG and its’ derived lncRNA SNHG17 on radiation-induced epithelial–mesenchymal transition (EMT) in vitro and RIPF in vivo. As revealed by high-throughput sequencing, SNHG17 expression was decreased by LGG treatment in A549 cells post radiation and markedly attenuated the radiation-induced EMT progression (*p* < 0.01). SNHG17 overexpression correlated with poor overall survival in patients with lung cancer. Mechanistically, SNHG17 can stabilize PTBP1 expression through binding to its 3′UTR, whereas the activated PTBP1 can bind with the NICD part of Notch1 to upregulate Notch1 expression and aggravated EMT and lung fibrosis post radiation. However, SNHG17 knockdown inhibited PTBP1 and Notch1 expression and produced the opposite results. Notably, A549 cells treated with LGG also promoted cell apoptosis and increased cell G2/M arrest post radiation. Mice of RIPF treated with LGG decreased SNHG17 expression and attenuated lung fibrosis. Altogether, these data reveal that modulation of radiation-induced EMT and lung fibrosis by treatment with LGG associates with a decrease in SNHG17 expression and the inhibition of SNHG17/PTBP1/Nothch1 axis. Collectively, our results indicate that LGG exerts protective effects in RIPF and SNHG17 holds a potential marker of RIPF recovery in patients with thoracic cancers.

**Supplementary Information:**

The online version contains supplementary material available at 10.1186/s13062-023-00357-x.

## Introduction

Ionizing radiation (IR)-induced lung fibrosis (RIPF) is a typical damage of the lung following radiotherapy for thoracic cancers [1]. The main characters of RIPF include progressive dyspnea, increasing accumulation of interstitial fluids and eventually the respiratory failure [2]. The incidence of RIPF is up to 20% of patients received radiation therapy [3]. Epithelial–mesenchymal transition (EMT), a morphologic switch from the epithelial polarized phenotype to the mesenchymal fibroblastoid phenotype, plays critical role in progress of RIPF [4]. The underlying mechanisms attributed to the EMT-derived RIPF have yet to be fully illustrated, effective prevention and protective strategies as well as underlying therapy targets also remain unclear.

SNHG17 identified as a long noncoding RNA (lncRNA), with lengths of 1186 nucleotides and its protein coding ability lost or restricted [5]. They found SNHG17 is highly overexpression in colorectal cancer, promoted cancer cell proliferation and is an unfavorable prognostic factor [6]. A recent review summarized SNHG17 is a novel cancer-related lncRNA which is highly overexpression in various cancers exerting oncogenic functions [7]. SNHG17 is also an EMT-related lncRNA with the evidence that TGF-beta1 activates SNHG17 expression, promoting cancer cells EMT, leading to the facilitation of esophageal squamous cell growth [8]. SNHG17 can promote lung adenocarcinoma EMT progression through sponging microRNA-193a-5p [9]. These previous studies show that the upregulation of SNHG17 exerts oncogenic functions through regulating EMT process. It may represent a promising therapeutic target in cancer therapy. However, whether the SNHG17 could regulate the RIPF via EMT remains incompletely clear.

PTBP1 (polypyrimidine tract-binding protein 1) belongs to the PTB family, known as critical regulator in posttranscriptional gene expression to mediate alternative splicing, translation, stability and localization [10]. Previous study has reported that lncRNA can regulate PTBP1 functions in breast cancer development [11], autophagy process [12] and inflammation activation [13]. It will be interesting to further investigate whether the PTBP1 can be regulated by active probiotics.

*Lactobacillus rhamnosus GG* (LGG), isolated from healthy human intestine, is one of the most widely supplied probiotics for dairy foods including yogurt and prefers beneficial living microorganisms for human health [14]. LGG was reported to not only protect the intestinal barrier, improving the diarrhea symptoms of patients with irritable bowel syndrome [15], but also has prevention and therapy benefits for inhibiting cancer formation [16], improving chemotherapy resistance [17] and protecting against ultraviolet radiation-induced carcinogenesis [18], X-ray-induced mice testis [19] and radiation-induced testis damage [20]. Recently, probiotic strains have been reported to be effective in health by regulating lncRNA expression. A study showed that lncRNASRD5A3 can be modulated by probiotic–prebiotic–synbiotic attenuated nonalcoholic steatohepatitis progression and ameliorated both of fibrosis and hepatic inflammation [21]. Another study showed the ability to slow the progression of non-alcoholic fatty liver disease by modulating lncRNA RPARP AS-1 [22]. These investigation suggest that the functions of LGG on health may be associated through regulation of non-coding RNAs. We hypothesized that in radiation-induced EMT, LGG may decline the expression of SMHG17, inhibiting its oncogenic functions, resulting in the prevention and protective role in inhibiting RIPF. Thus, we aimed to investing whether the SNHG17 expression can be regulated by LGG and the effects of SNHG17 deficiency on the radiation-induced cell response.

## Results

### SNHG17 is a LGG-modulated lncRNA in response to radiation

**Fig. 1:**
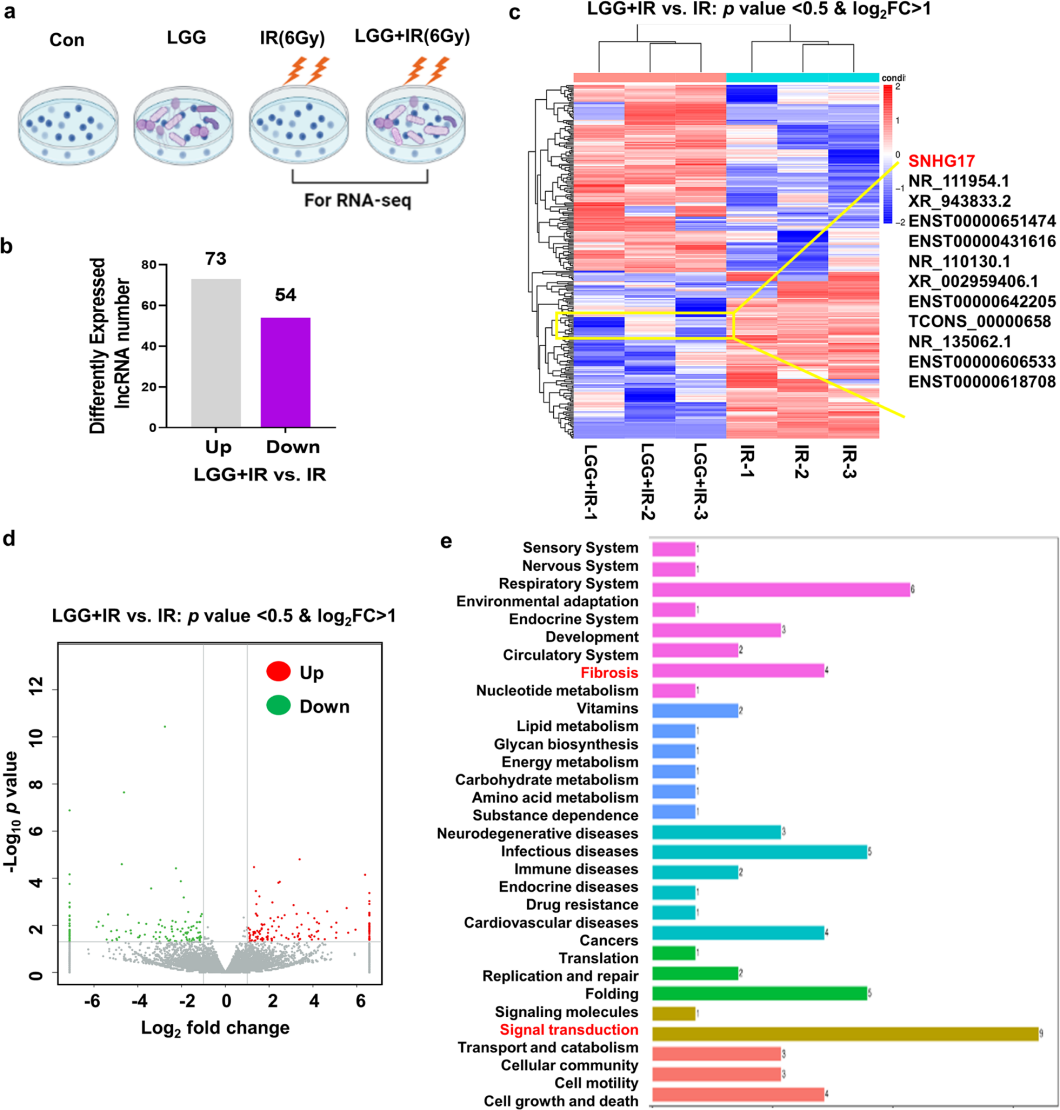
SNHG17 is a LGG-modulated lncRNA in response to radiation. **a** A549 cells were divided into 4 groups, control group (Con), treated with LGG group (LGG), 6 Gy radiation group (IR) and LGG treated prior to 6 Gy radiation (LGG + IR). **b** Up- and down-regulated numbers of lncRNAs in LGG + IR versus IR. **c** Heatmap of differentially expressed lncRNAs in LGG + IR versus IR. Red presents upregulated lncRNAs and purple presents down-regulated lncRNAs. **d** Volcano plot of differentially expressed lncRNAs in LGG + IR versus IR. Red presents upregulated lncRNAs and green presents down-regulated lncRNAs. **e** KEGG analysis the predicted pathway involved by the differential expressed lncRNA within LGG + IR versus IR. Data are means ± SD (standard deviation). N = 3 independent experiments, Student’s two-tailed unpaired t test was used to compare differences between two groups. *p < 0.05

To explore the potential lncRNAs involved into LGG modulation of lung cancer cells post radiation, we analyzed lncRNAs prolife in A549 cells with or without LGG supplement post 6 Gy radiation. Three samples of LGG-negative A549 cell and three samples of LGG-positive cells were collected for the microarray analysis of lncRNAs (Fig. 1a). After screening, 73 lncRNAs expression increased whereas 54 lncRNAs expression decreased in LGG treatment group with compared to non-LGG treatment group post 6 Gy radiation (Fig. 1b). Cluster analysis and Volcano assay illustrated that the amount of increased lncRNAs were more than decreased lncRNAs (Fig. 1c, d). Further Kyoto Encyclopedia of Genes and Genomes (KEGG) bioinformatics assay showed these changed lncRNAs enriched in regulation of circulatory system function, lipid metabolism function, in particular, the regulation of fibrosis and signal transduction (Fig. 1d), indicating these lncRNAs may potential for performing critical role in the biological function after LGG modulation in response to radiation. Among these changed lncRNAs, SNHG17 showed the highest decrease expression level after LGG treatment in A549 cells as compared to the other lncRNAs (Additional file 1: Table S1). These results indicated that SNHG17 is a LGG-modulated lncRNA and can response to radiation.

### SNHG17 expression increases in lung cancer tissues and serves as a poor prognosis biomarker

As previous research studies have found SNHG17 is an oncogenic lncRNA and participant in multiple cancer regulation-associated signaling pathway [5], we focused on exploration its function from LGG modulation perspective. We first conducted bioinformatics assay based on The Cancer Genome Atlas (TCGA) database and online GEPIA database (http://gepia.cancer-pku.cn/). SNHG17 is frequently upregulated in LUAD (lung adenocarcinoma) and LUSC (lung squamous cell carcinoma) tissues compared with their paired non cancer tissues (Additional file 1: Fig. S1A, B). Furthermore, SNHG17 expression levels were positively correlated with cancer process stage (Additional file 1: Fig. S1A, B). The Kaplan–Meier method and log-rank test assay demonstrated that patients with high SNHG17 expression levels exhibited poor overall survival in patients with LUSC, ACC (Adrenocortical carcinoma), COAD (Colon adenocarcinoma) and LIHC (Liver hepatocellular carcinoma) (Additional file 1: Fig. S2E–H), respectively. We validated the overexpression of SNHG17 in LUAD cohort 1 (cancer tissues = 15, adjacent normal tissues = 15) and LUSC cohort 2 (cancer tissues = 15, adjacent normal tissues = 15) by qRT-PCR and found SNHG17 was upregulated in LUAD and LUSC tissues compared with their paired adjacent normal tissues (Additional file 1: Fig. S1I, J). These results indicated that SNHG17 may potential for lung cancer progress and prognosis.

### SNHG17 expression is decreases by LGG treatment in lung cancer cells post radiation

As of the reported oncogenic property of SNHG17, we further validated that SHNG17 expression increased in A549 and H1299 cancer cells compared with normal lung epithelial cell, HBE and BEAS2B cells (Additional file 1: Fig. S2A). We also found the SNHG17 expression increased post radiation in a dose- and time-dependent manner (Additional file 1: Fig. S2B, C). To examine the effect of LGG on SNHG17 expression, we co-culture A549 cells with LGG and found SNHG17 expression decreased with LGG in a dose-dependent manner (Additional file 1: Fig. S2C). To evaluate whether the effect of decreasing SNHG17 expression is specific to LGG, we compared the modulation ability within viable LGG and ethanol-killed LGG. The result showed only viable LGG efficiently modulated SNHG17 expression (Additional file 1: Fig. S2D). To investigate the combination role of radiation and LGG, we co-culture the A549 cells with or without LGG and 6 Gy radiation. The phenotype of cells is illustrated in Additional file 1: Fig. S2F. SNHG17 expression increased post radiation but decreased after LGG treatment post radiation (Additional file 1: Fig. S2G). IF assay further demonstrated that SNHG17 expression decreased after LGG treatment post radiation (Additional file 1: Fig. S2H). These results indicated that LGG has the ability to decrease SNHG17 expression post radiation.

### SNHG17 deficiency attenuates the radiation-mediated cell proliferation, apoptosis and G2/M transition

**Fig. 2:**
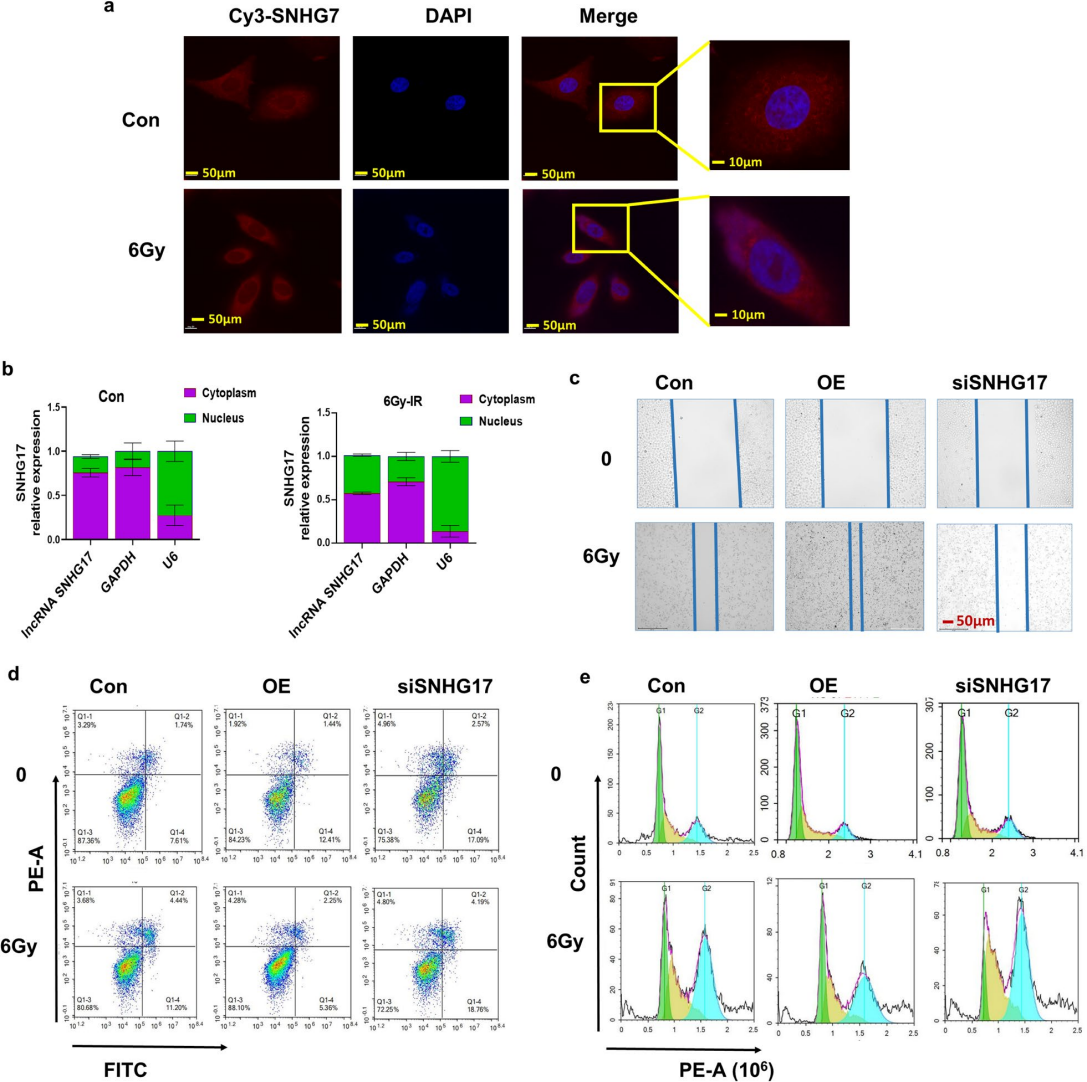
SNHG17 transfers from cytoplasm to nucleus post radiation. **a** Representative photographs of the location of SNHG17 in cells prior and post radiation through Fluorescence in situ hybridization (FISH) assay by confocal microscopy. **b** Quantitative analysis of SNHG17 expression in cytoplasm and nucleus prior and post radiation through nucleocytoplasmic separation assay. **c** Representative photographs of migration ability after knockdown SNHG17 expression in A549 cells with or without 6 Gy treatment through scratch wound healing migration assay. **d** Representative photographs of cell apoptosis at 24 h after knockdown SNHG17 expression in A549 cells with or without 6 Gy treatment. **e** Representative photographs of cell cycle after knockdown SNHG17 expression in A549 cells with or without 6 Gy treatment through flow cytometric cell cycle assay. GAPDH served as the cytoplasmic expression control and U6 severed as the nuclear expression control. Error bars represent the SEM of the mean of 3 independent experiments. Data are means ± SD (standard deviation). N = 3 independent experiments, Student’s two-tailed unpaired t test was used to compare differences between two groups. *p < 0.05

The results of LGG-modulated lncRNA post radiation KEGG assay revealed that fibrosis and signaling transduction pathway were the most enriched pathway, indicating that SNHG17 may involve in the radiation-mediated biological function. First, we used SNHG17 probe and found SNHG17 translocated from cytoplasm to nuclear after radiation (Fig. 2a). Nucleocytoplasmic separation experiment further confirmed the SNHG17 expression increased in nucleus after radiation (Fig. 2b). Knockdown SNHG17 by siRNA inhibited A549 cancer cells’ migration ability whereas overexpression SNHG17 expression enhanced cell migration post radiation (Fig. 2c). Also, knockdown SNHG17 expression promoted cell apoptosis whereas overexpression SNHG17 expression inhibited cell apoptosis at 24 h post radiation (Fig. 2d). Cell cycle detection illustrated that knockdown SNHG17 expression promoted G2/M arrest whereas overexpression SNHG17 expression promoted cell G2/M transition at 2 h post radiation (Fig. 2e).

### SNHG17 deficiency attenuates the radiation-mediated EMT progress

We then analyzed the SNHG17 role in regulation radiation-induced EMT (epithelial–mesenchymal transition) in normal BEAS2B cells. First, post radiation, E-caherine, a key biomarker of epithelial decreased and N-cadherine, a key biomarker of mesenchymal increased in a dose- and time-dependent manner (Additional file 1: Fig. S3A, B). While treated with LGG, the E-cadherine expression switched to increase and Vimentin, N-cadherin and a-SMA expression switched to decrease post radiation indicating the protection function of LGG in the cells response to radiation (Additional file 1: Fig. S3C). However, knockdown SNHG17 increased E-cadherine expression but decrease Vimentin expression post radiation in compared without siRNA treatment, whereas overexpression of SNHG17 decreased E-cadherine expression but increase Vimentin expression post radiation (Additional file 1: Fig. S3E). Similarly, the effects of SNHG17 on EMT could be repeated in another normal lung epithelial cell line, HBE (Additional file 1: Fig. S3E).

### SNHG17 can bind with PTBP1 post radiation directly

**Fig. 3:**
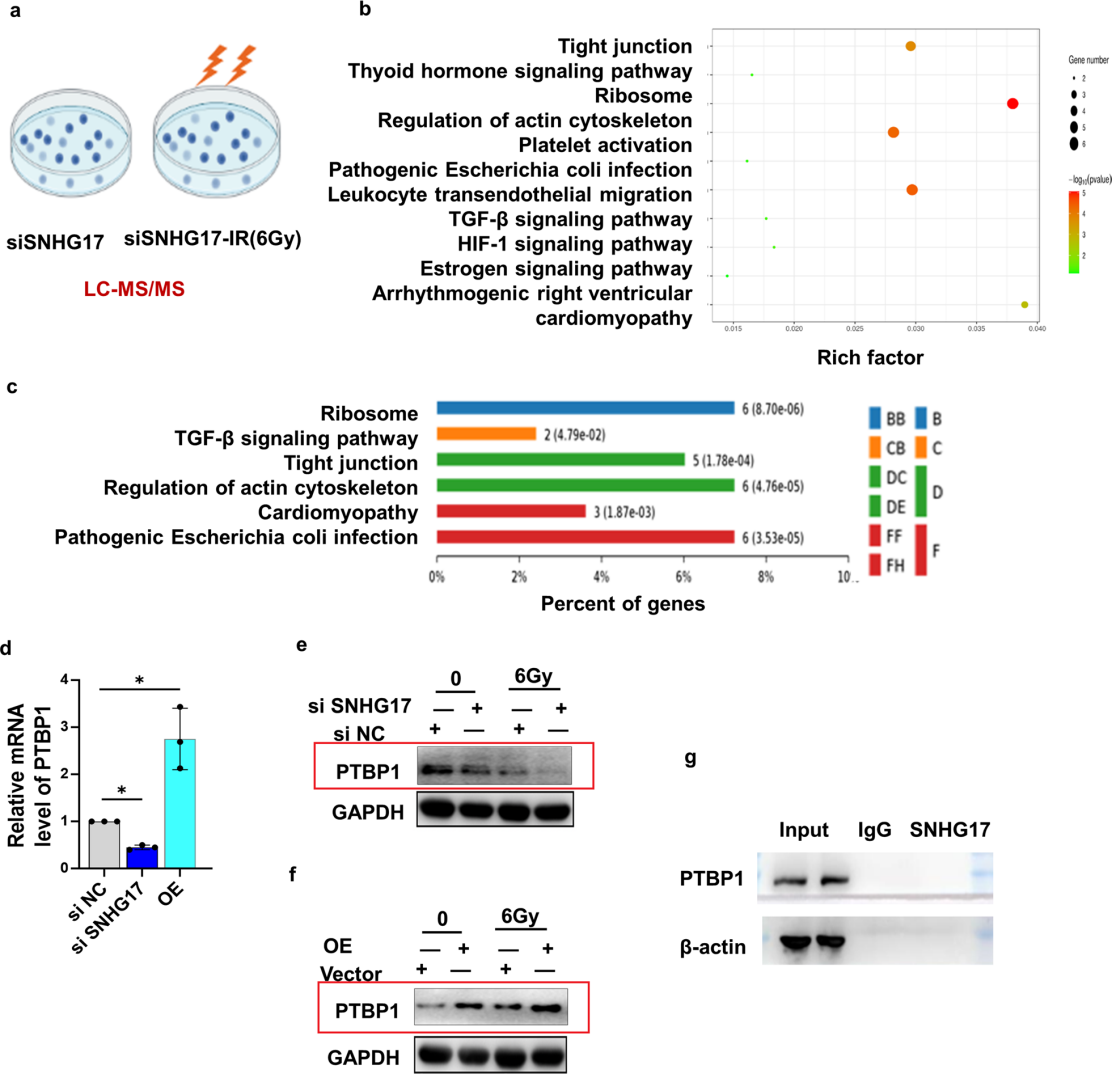
SNHG17 can interact with PTBP1 post radiation. **a** A549 cells were divided into 2 groups, knockdown of SNHG17 group (siSNHG17), treated with IR followed by knockdown of SNHG17 group (siSNHG17-IR). Cells were collected after treatment and send to BiotechPack Seientific Ltd. Beijing, China for LC–MS/MS detection to discover potential SNHG17-interacted proteins. **b** GO assay of the potential biological function for differential binding proteins. **c** KEGG assay of the potential biological pathway for differential binding proteins. **d** Quantitative analysis of PTBP1 mRNA expression in knockdown and overexpression of SNHG17 in A549 cells by qRT-PCR assay. **e** Representative blots of PTBP1 protein expression in knockdown of SNHG17 in A549 cells post 6 Gy radiation by Western blotting assay. **f** Representative blots of PTBP1 protein expression in overexpression of SNHG17 in A549 cells post 6 Gy radiation by Western blotting assay. **g** RNA pull-down analysis of the binding of SNHG17 with PTBP1 in total protein extracted from A549 cells post radiation. Data are means ± SD (standard deviation). n = 3 independent experiments, Student’s two-tailed unpaired t test was used to compare differences between two groups. *p < 0.05

As of the critical role of SNHG17 in regulation of cells in response to radiation, we next explored the possible molecular mechanism. A549 cells with siSNHG17 were treated with or without 6 Gy and potential binding proteins were detected by liquid chromatography–mass spectrometry (LC/MS) (Fig. 3a). Typically, 69 protein complexes were upregulated and 47 were downregulated (Additional file 1: Table S2). Of these changed proteins, PTBP1 ranks at the top 1 down-regulation protein. A Gene Ontology (GO) assay showed that these RNA–protein complexes were involved in the regulation of tight junction, ribosome, and TGF-β signaling pathway (Fig. 3b). A KEGG analysis showed that these RNA–protein complexes were enriched in ribosome or TGF-β signaling pathway (Additional file 1: Fig. S3C). Knockdown of SNHG17 expression was positive associated with PTBP1 mRNA expression whereas overexpression SNHG17 was negative associated with PTBP1 mRNA expression (Fig. 3d). Further, PTBT protein expression decreased in cells treated with siSNHG17 but increased in cells treated with overexpression SNHG17 post radiation (Fig. 3e, f), indicating SNHG17 may positively regulate PTBP1 mRNA and protein expression post radiation. RNA pull-down showed that SNHG17 has a binding ability to the PTBP1 (Fig. 3g).

### SNHG17 stabilizes the PTBP1 expression by binding to its 3′UTR

**Fig. 4:**
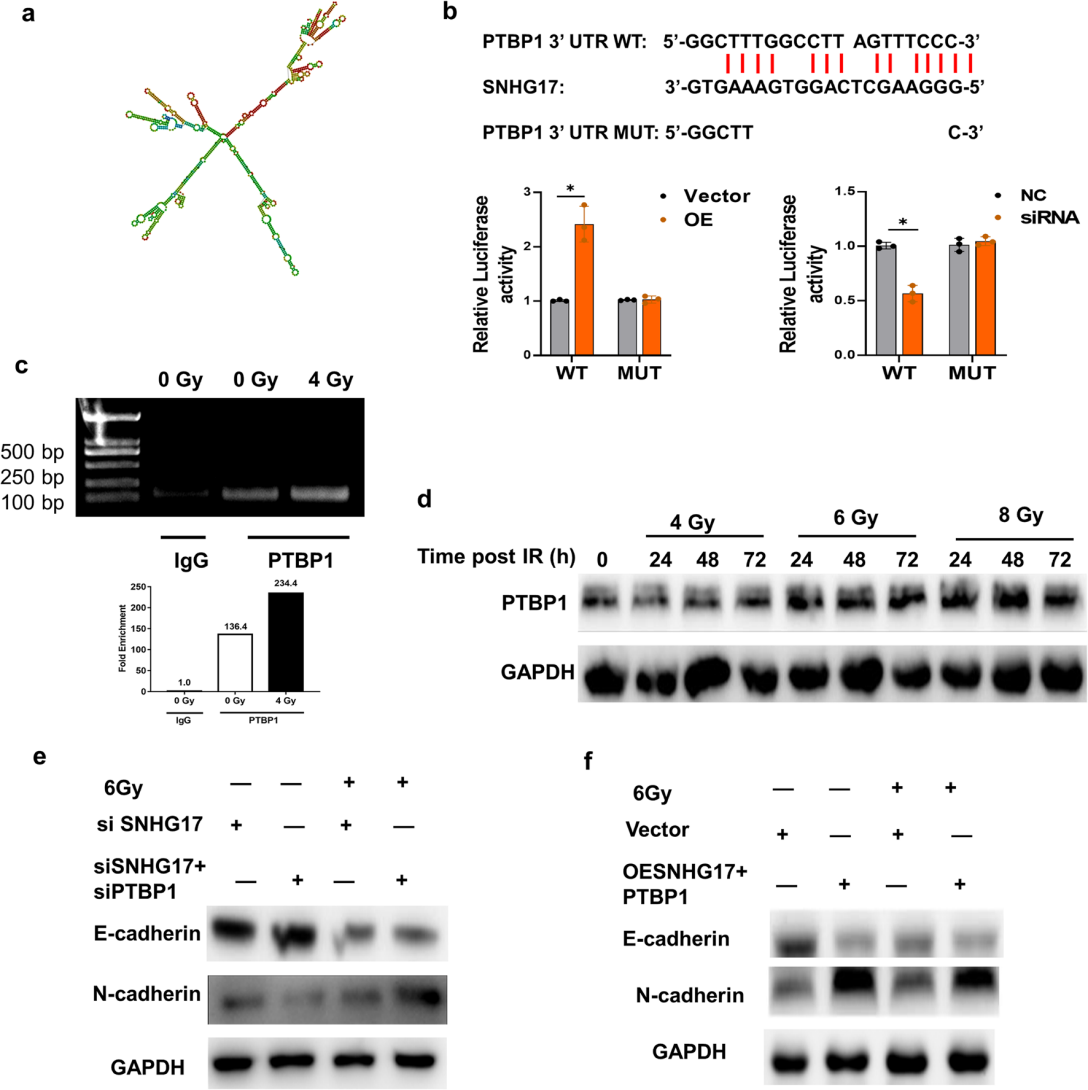
SNHG17 stabilizes the PTBP1 expression by binding to its 3′UTR. **a** Structural analysis using an online tool (http://rna.urmc.Rochester.edu/RNAstructureWeb/). **b** Bioinformatic information analysis predicted SNHG17 and PTBP1 binding sites in SNHG17 sequence. The transcript activity of PTBP1 was measured in A549 cells after knockdown of SNHG17 or overexpression of SNHG17. **c** Immunoblot assay of PTBP1 and β-actin in the RNA pulldown extract with biotin-labeled full-length lncRNA SNHG17 by RT-PCR. Biotin-anti-sense lncRNA SNHG17 sequences were used as negative control. **d** PTBP1 protein expression at indicated radiation dosage and timepoints of radiation by Western blotting. **e** Representative blots of E-cadherin and N-cadherin protein expression in knockdown of SNHG17 or knockdown of PTBP1 in A549 cells post 6 Gy radiation by Western blotting assay. **f** Representative blots of E-cadherin and N-cadherin protein expression in overexpression of SNHG17 or overexpression of PTBP1 in A549 cells post 6 Gy radiation by Western blotting assay. Data are means ± SD (standard deviation). n = 3 independent experiments, Student’s two-tailed unpaired t test was used to compare differences between two groups. *p < 0.05

SNHG17 structural data obtained through RNA structure analysis is presented online (http://rna.urmc.Rochester.edu/RNAstructureWeb/) (Fig. 4a). Additionally, through UCSC (University of California Santa Cruz Genome Brower) database screening and bioinformatics binding sequence to the 3′UTR of PTBP1 mRNA (Fig. 4b). The dual-luciferase reporter gene assay showed that SNHG17 can regulate the luciferase activity of the PTBP1 3′UTR (Fig. 4b). The RIP assay further confirmed that SNHG17 binds to the 3′UTR of PTBP1 mRNA in A549 cells post radiation (Fig. 4c). PTBP1 expression increased post radiation in a time- and dose-dependent manner (Fig. 4d). Knockdown of both of SNHG17 and PTBP1 increased E-cadherin expression and decreased N-cadherin expression than that of only knockdown of SNHG17 expression post radiation (Fig. 4e), while overexpression of both of SNHG17 and PTBP1 expression provided the opposite result in A549 cells post radiation (Fig. 4f), indicating SNHG17 can stabilize PTBP1 expression through bind to the 3′UTR.

### PTBP1 combines with Notch1 post radiation

**Fig. 5:**
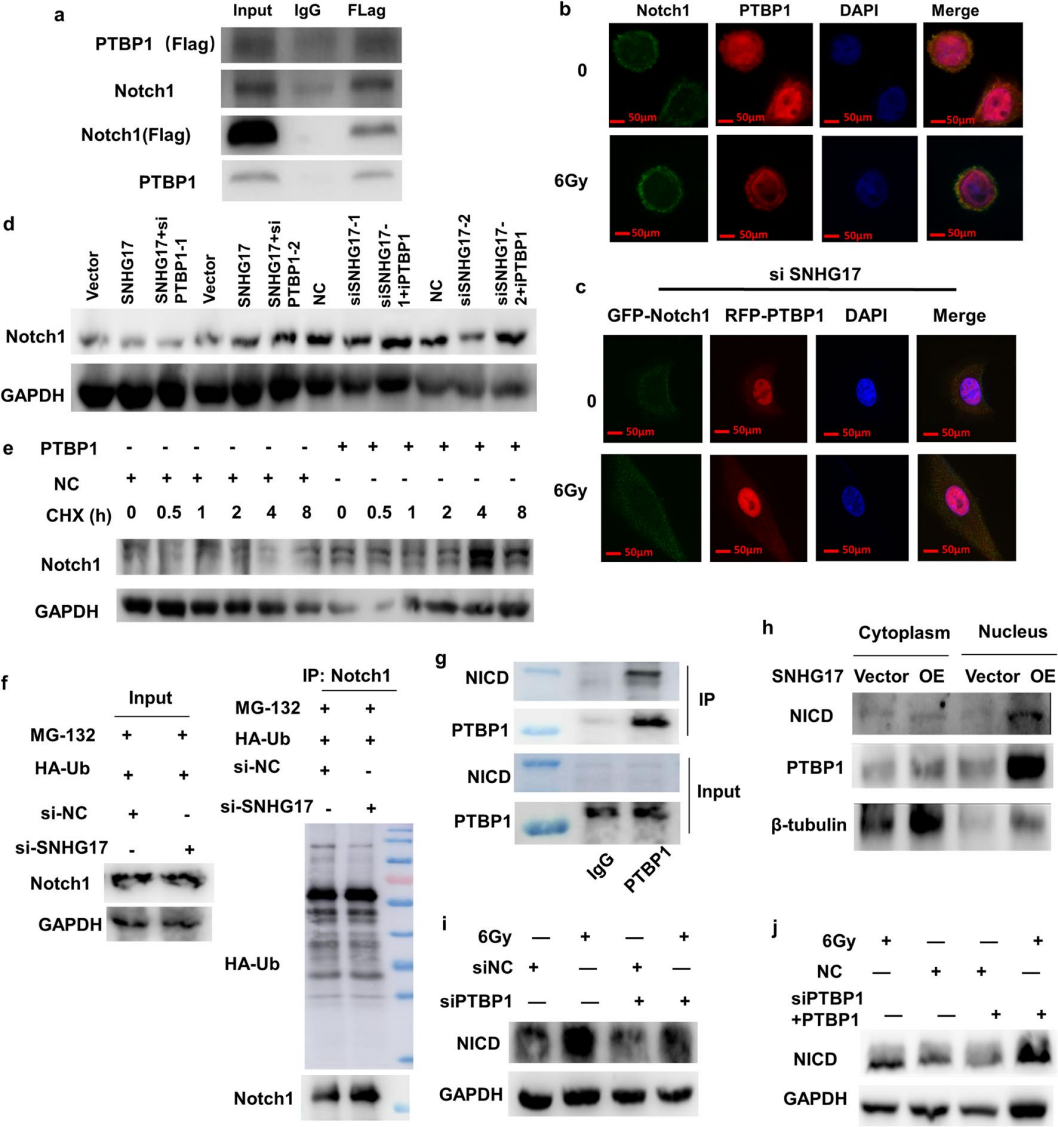
PTBP1 interacts with NICD to activate Notch1 expression. **a** RIP assay was shown the interaction between PTBP1 and Notch1. **b** Co-localization of PTBP1 and Notch1 protein in A549 cells post radiation was determined by immunofluorescence. Scale bars = 50 μm. **c** Co-localization of PTBP1 and Notch1 protein in A549 cells with or without knockdown SNHG17 expression post radiation was determined by immunofluorescence. Scale bars = 50 μm. **d** The Notch1 protein expression was measured after overexpression SNHG17 or PTBP1, or after knockdown of SNHG17 or PTBP1 was determined by Western blotting. **e** Representative images of the Western blotting results of the Notch1 protein in si NC and si PTBP1-transfected A549 cells after indicated CHX(cicloheximide) treated timepoints (0, 0.5, 1, 2, 4, 8 h). **f** Immunoprecipitation analysis of ubiquitinated Notch1 in si NC and si SNHG17-transfected A549 cells pretreated with the proteasome inhibitor MG132. **g** Immunoprecipitation analysis of PTBP1 and NICD interaction. **h** The nuclear NICD and PTBP1 expression was examined in A549 cells after overexpression of SNHG17 by western blotting. **i** Representative blots of NICD protein expression in knockdown of PTBP1 in A549 cells post 6 Gy radiation by Western blotting assay. **j** Rescue assay was shown NICD protein expression in overexpression of PTBP1 following knockdown of PTBP1 in A549 cells post 6 Gy radiation by Western blotting assay. Data are means ± SD (standard deviation). n = 3 independent experiments, Student’s two-tailed unpaired t test was used to compare differences between two groups. *p < 0.05

To identify the possible function of PTBP1 on A549 cells regulated by SNHG17, Hitpredict and GenMaNIA databases were used to identify the potential proteins with PTBP1. Here, we selected Notch1 because it was reported a key pathway involving in the EMT progress and also the predicted protein interacted with PTBP1 in both of two databases. Immunoprecipitation analysis indicated that PTBP1 could bind to Notch1 in A549 cells (Fig. 5a). Immunofluorescence indicated the co-localization within PTBP1 and Notch1 post radiation (Fig. 5b), while this interaction attenuated in cells treated with siSNHG17 post radiation, indicating the interaction within PTBP1 and Notch1 can be regulated by SNHG17 post radiation (Fig. 5c). Western blotting showed that Notch1 expression can be regulated by SNHG17 and PTBP1 (Fig. 5d). As of cicloheximide (CHX) can inhibit protein synthesis, we next analyzed the effects of PTBP1 on Notch1 protein stability in A549 cells. The results showed that PTBP1 could inhibit the degradation of the Notch1 protein (Fig. 5e). As of ubiquitination is an important type of protein degradation, we next detected ubiquitination of PTBP1 in A549 cells with or without siSNHG17. The results showed knockdown of SNHG17 could not ubiquitinate Notch1 protein (Fig. 5f). Considering NICD is the Notch1’s transcription activity protein fraction with function of regulation of EMT-related genes expression, we than conducted RIP assay and the results showed PTBP1 can bind to NICD (Fig. 5g). Overexpression of SNHG17 can increase NICD and PTBP1 expression in nucleus (Fig. 5h). Western blotting showed knockdown PTBP1 expression decreased NICD expression post radiation (Fig. 5i) whereas rescue assay further confirmed the role of PTBP1 in regulation of NICD (Fig. 5j). These results indicated that PTBP1 can bind with NICD and SNHG17 involved into regulation PTBP1-NICD interaction.

### LGG improves the radiation-induced cell proliferation and EMT

**Fig. 6:**
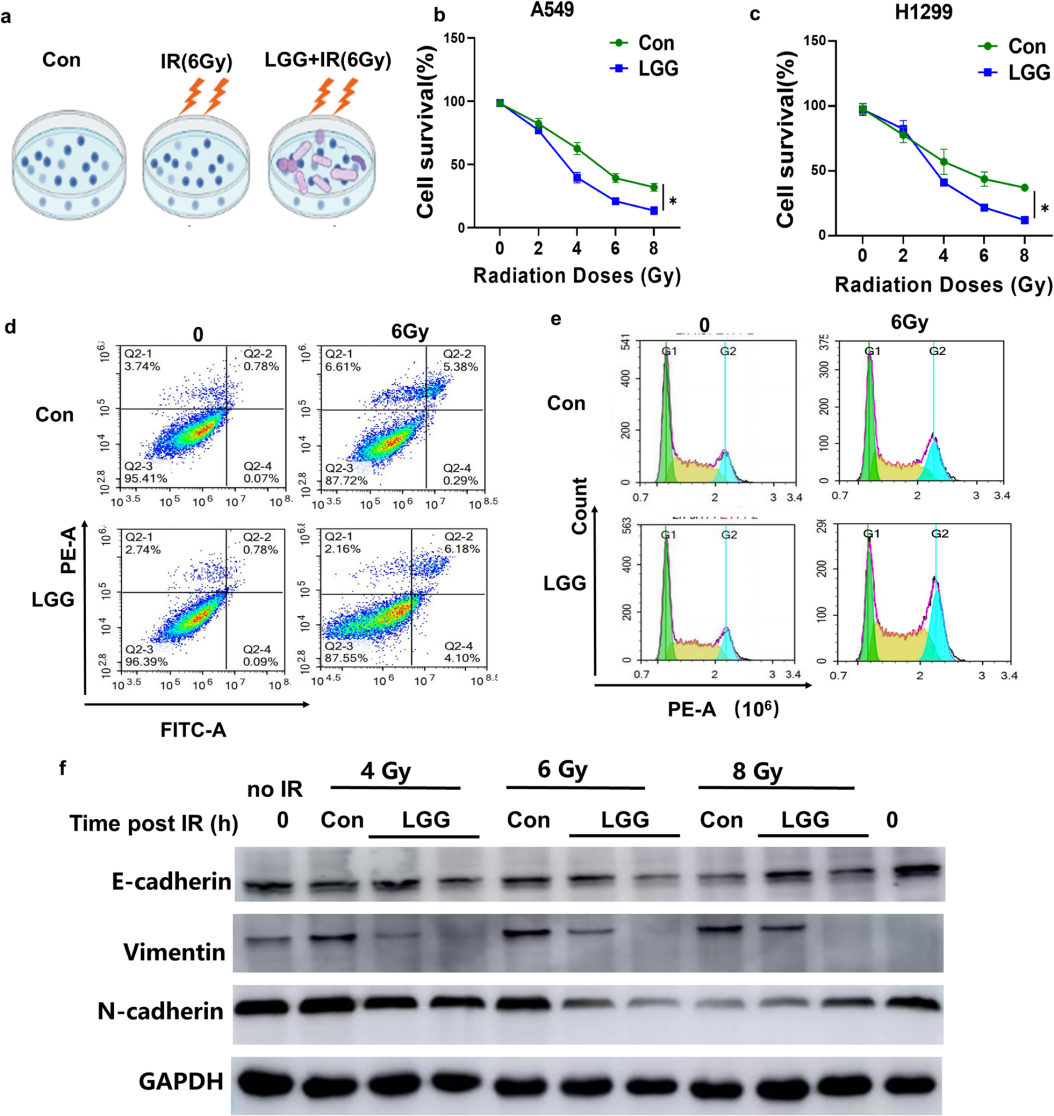
LGG improves radiation-induced lung EMT. **a** BEAS2B cells were divided into 3 groups, Control group (Con), 6 Gy radiation group (IR), treated with IR followed by LGG treatment group (LGG + IR). **b** Cell survival rate in Con and LGG groups post indicated dosage of radiation in A549 cells. **c** Cell survival rate in Con and LGG groups post indicated dosage of radiation in H1299 cells. **d** Representative photographs of cell apoptosis after LGG-treated in BEAS2B cells with or without 6 Gy treatment through flow cytometric assay. **e** Representative photographs of cell cycle after LGG-treated in BEAS2B cells with or without 6 Gy treatment through flow cytometric assay. **f** Representative blots of E-cadherin, VImentin and N-cadherin protein expression in LGG treated BEAs2B cells post 6 Gy radiation by Western blotting assay. Data are means ± SD (standard deviation). n = 3 independent experiments, Student’s two-tailed unpaired t test was used to compare differences between two groups. *p < 0.05

Since LGG modulated the decrease of SNHG17 expression in lung cancer cells post radiation, we then explore the effects of LGG on radiation-induced cell proliferation and EMT. A549 cells were divided into con, radiation and LGG treatment prior radiation groups (Fig. 6a). Compared with control group, LGG inhibited cell survival post radiation in A549 and H1299 cells (Fig. 6b, c). A549 cells treated with LGG also promoted cell apoptosis and increased cell G2/M arrest post radiation (Fig. 6d, e). Western blotting showed that LGG treatment increased E-cadherin expression and decreased VImentin, N-cadherin expression in BEAS2B cells post radiation (Fig. 6f) showing the protection of LGG on cells in response to radiation insults. These results indicate that LGG improves the radiation-induced cell proliferation and EMT.

### LGG-modulated SNHG17 deficiency improves the radiation-induced EMT in vivo

**Fig. 7:**
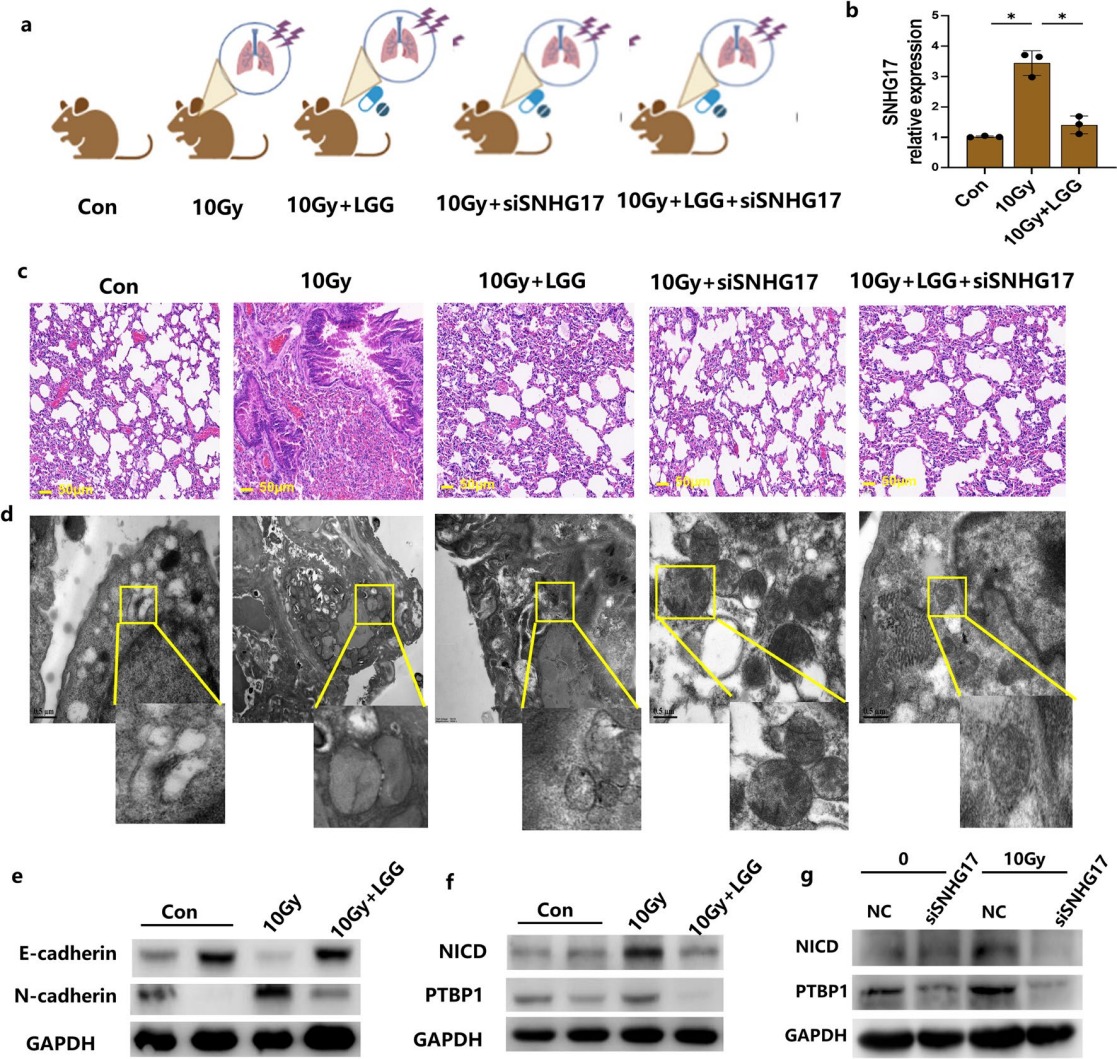
LGG and siSNHG17 improve radiation-induced lung fibrosis. **a** Male C57BL/6 mice were divided into five groups: control group (Con), radiation-induced lung fibrosis group with 10 Gy radiation treated once time (10 Gy), mice treated with LGG prior to received 10 Gy radiation on lung (10 Gy + LGG), mice treated with siSNHG17 through tail vein injection prior to received 10 Gy radiation on lung (10 Gy + siSNHG17), mice treated with LGG and siSNHG17 prior to received 10 Gy radiation on lung (10 Gy + LGG + siSNHG17). The mice were killed at the 7th weeks to collect tissues for further detection of various parameters. **b** SNHG17 expression in lung tissues detected by qRT-PCR. **c** Representative images of hematoxylin and eosin-stained metastatic lung tissues. Scale bar = 50 μm. **d** Representative photographs of lung tissues by TEM. **e** Representative blots E-cadherin, and N-cadherin protein expression in lung tissues with or without LGG treatment post 10 Gy radiation by Western blotting assay. **f** Representative blots NICD, and PTBP1 protein expression in lung tissues with or without LGG treatment post 10 Gy radiation by Western blotting assay. **g** Representative blots NICD, and PTBP1 protein expression in lung tissues with or without knockdown of SNHG17 post 10 Gy radiation by Western blotting assay. Data are means ± SD (standard deviation). n = 3 independent experiments, Student’s two-tailed unpaired t test was used to compare differences between two groups. *p < 0.05

To investigate the SNHG17 role in vivo, we further evaluated the effect of LGG and SNHG17 silencing on radiation-induced lung EMT progression in vivo. Mice were divided into 5 groups as shown in Fig. 7a. A mouse model of radiation-induced lung fibrosis was established via a single dose of 10 Gy to the whole thorax. SNHG17 mRNA expression increased post radiation in lung tissues but decreased in lung tissues in mice treated prior with LGG (Fig. 7b). HE staining showed at 12 weeks after 10 Gy radiation, the lung tissue damaged with thickened alveolar septa, interstitial oedema and infiltrated inflammatory cell were obviously mitigated, but in LGG and siSNHG17 treated mice, these damage was attenuated and the lung alveolar integrity was better than that in radiation-induced lung fibrosis group (Fig. 7c). TEM showed that the mitochondrion structure was damaged but restored after LGG and siSNHG17 treatment (Fig. 7d). Western blotting showed LGG increased E-cadherin expression and decreased N-cadherin expression in mice lung tissues post radiation (Fig. 7e). Also, LGG decreased NICD and PTBP1 expression in mice lung tissues post radiation (Fig. 7f). SNHG17 deficiency decreased NICD and PTBP1 expression in mice lung tissues post radiation (Fig. 7g). These results indicate that LGG may act as protection role in inhibiting radiation-induced EMT in vivo.

## Discussion

The RIPF is a severe side effect in patients with thoracic associated cancer after radiotherapy. Future research should be paid more attention to explore the prevention or protective targets and strategies of RIPF. Recent studies have shown that epigenetic modification including lncRNAs are thought to be critical in the interaction between the radiation exposure and damage [23]. Furthermore, the probiotics have been identified to prevent the damage of radiation [24]. Both lncRNAs and probiotics have important health and disease roles. As a result, study on how probiotics regulate lncRNAs would provide new insight for controlling the radiation-induced damage which could be used as a new approach to prevention and therapy of RIPF. Our study revealed that SNHG17, identified by LGG treated lung cancer cells post radiation through RNA-seq analysis, is associated with RIPF and promote EMT progression. We focused on the LGG-derived lncRNA because of the widely used of LGG but limited associated molecular mechanism illustration in epigenetic aspect, in particular, the mechanism of the protection role of LGG in lung cancer patients with radiation-induced fibrosis through regulation of lncRNA.

Upon to LGG, it is typically a widely used and characterized probiotic train in health promotion. Recently, probiotics including LGG has been used for improving cancer chemo- and radiotherapy, to relieve adverse symptoms or increase quality of life [25]. Evidence has shown that the health effects of probiotics are associated with specific genes and even individual nucleotides, but the relationship between epigenetic regulation of probiotics and health remains poorly understood. Demont et al. found both live and heat-treated probiotics can modulate miRNA expression [26]. Xing et al. found that *Bacillus coagulans R11* can decrease intestinal injury induced by lead exposure through affecting the faecal miRNA functions [27]. Chen et al. found that *Lactobacillus plantarum Z01* can reduce *Salmonella* Typhimurium-induced cecal inflammation through regulating miRNA expression [28]. A recent review showed the crosstalk between miRNA and probiotics may influence the development of inflammatory bowel disease pathogenesis and therapeutics [29]. Hany et al. showed probiotics can slow the NAFLD progression through regulation of lncRNA RPARP AS-1 [22], whereas Gadallah et al. showed probiotics can slow the progression of NAFLD through regulation of lncRNASRD5A3-AS1 [21]. Since miRNAs and lncRNAs dysregulation plays an important role in the pathogenesis of many different diseases, these reports indicate that the mechanism of probiotics effects on disease therapy may be associated with epigenetic regulation. However, most of previous reports focused on the effects of probiotics on miRNAs instead of the effect of signal probiotics strain. Moreover, the role of LGG-lncRNA interactions in radiotherapy has limited reports. The novelty of our finding showed that LGG can modulate lncRNA expression in cancer cells. This provides a new avenue that probiotics involve into epigenetic regulation and a deep understanding of probiotics-lncRNA cross-talk for further better application in cancer therapy.

Upon to SNHG17, it has been identified as a novel long-coding RNA, referring to oncogenic function in various cancers[7]. SNHG17 has high expression in many cancer tissues and can be induced by many factors including m6A methyltransferase METTL3 [30]. The molecular mechanisms of SNHG17 mainly rely on the ceRNA or interact with downstream proteins directly, resulting in promotion of cancer proliferation, growth or migration or serving as poor prognosis predictor [31, 32]. In our results, we found SNHG17 can be modulated by LGG. Further study showed SNHG17 expression increased post radiation but decreased following LGG treatment prior radiation. Moreover, we found SNHG17 overexpression can promote radiation-induced EMT. These results are consistent with previous reports that SNHG17 is an oncogenic molecular and our data provide more information regarding the role of SNHG17 in cancer cells response to radiation. Most importantly, we found SNHG17 can bind the 3′UTR of PTBP1 to stabilize its expression to activate Notch1 expression. Upon to PTBP1, it has been reported as a splicing suppressor, competing with spliceosome for RNA binding to inhibit alternative splicing [11]. PTBP1 has also been reported to promote EMT progression in breast cancer [33] and in gastric cancer [13]. Miao et al. found that lncRNA MALAT1 can stabilize the interaction between PTBP1 and other proteins to affect the alternative splicing events [34]. Another study indicated that lncRNA LINREP can promote glioblastoma progression through recruiting the formation of PTBP1/HUR complex [35]. Notch1 is a key transcription factor in regulation of lung fibrosis [36]. The inhibition role of LGG on decreasing SNHG17 expression may inactivate PTBP1 and Notch1 expression leading to the attenuation of RIPF. In general, our study reveals the role of LGG/SNHG17/PTBP1 in attenuating lung cancer cell proliferation and EMT progression, and suggests that SNHG17 may serve as a novel therapeutic target for RIPF.

Upon to the prevention and therapy of EMT and lung fibrosis, Lactobacillus was reported to attenuate the pancreatic cancer EMT progression [37] and enhanced the 5-FU anticancer activity in colorectal cancer [38]. Our results further found that LGG can inhibit radiation-induced EMT and RIPF, providing the new potential application of LGG in prevention or therapy of RIPF in clinic.

In summary, SNHG17 was markedly deregulated in LGG treated lung cancer A549 cells in response to radiation. Moreover, SNHG17 is an lncRNA that promotes radiation-induced EMT and lung fibrosis through stabilizing PTBP1 expression and activating Notch1 expression. Our results demonstrate that SNHG17 is not only a potential biomarker for early diagnosis and treatment of radiation-induced EMT and RIPF, but also provides baseline understanding of how the contributing effect of LGG on radiation-induced EMT and RIPF through epigenetic regulation mechanism. Downregualtion of SNHG17 by LGG appears to potential prevention target and explains at least one of missing links among probiotics, epigenetic regulation and cancer therapy.

## Materials and methods

### Cell lines and irradiation condition

Human non-small cell lung cancer A549 cell line, human lung epithelial HBE cell line and BEAS2B cell, human renal epithelial 293T cells were stored in our laboratory and authenticated by ATCC (American Type Culture Collection, USA). A549, BEAS2B and 293T cells were maintained in DMEM (HyClone) with 10% fetal bovine serum (Gibco, Scoresby, Victoria, Australia). In addition, 1% penicillin streptomycin-glutamine was added to the medium, and the cells were maintained at 37 °C in a humidified incubator containing 5% CO_2_.

γ-Radiation was performed using a ^60^Co source at the Institute of Radiation Medicine, Academy of Military Medical Sciences (Beijing, China). A549 and H1299 cells were sham-radiated or exposed to radiation at 6 Gy (dose rate: 127.15 cGy/min, source distance: 70 cm, voltage: 180 kV, current: 12.5mA). The cells were irradiated with ^60^Co γ-rays at room temperature as our team previous reports [4, 39]. To explain whether there exerted dose- and time-dependent manner, A549 cells were subjected to ^60^Co γ-rays irradiation at 4 Gy, 6 and 8 Gy, respectively (dose rate: 127.15 cGy/min, source distance: 70 cm, voltage: 180 kV, current: 12.5 mA) as our team previous reported [40–42].

### Cell transfection

The cells were passaged the day before transfection. After the cells were grown to 60% density, siRNA (the sequence are listed in Additional file 1: Table S1) was conducted through the transient transfection using Lipofectamine 2000 (Thermo Fisher Scientific, Waltham, MA, USA) following the manufacturer’s instructions. Scrambled siRNA was used as the negative control. Forty-eight hours after transfection, the cells were collected for further experiments.

### Cell proliferation analysis

For the cell proliferation analysis, cells were collected at passage 3–4, and inoculated in 96-well plates at a density of 3 × 10^3^ cells/well. The effects of LGG on cell viability were detected using a standard cell counting kit-8 (CCK-8) according to the manufacturer’s instructions (CK04, DOJINDO, Japan). The optical density (OD) of the cells in each group was tested by measuring absorbance at 570 nm using a microplate reader.

### Establishment of co-culture of cells and LGG

Prepared a concentration of 3.0 × 10^6^ cells/ml cell suspension in the six well cell culture plate, discard the cell culture medium, add PBS solution for three times, and then put the 0.4 µM Transwell in the well of the culture plate. Then each well was divided into the upper chamber and the lower chamber. The upper chamber is the upper chamber, and the lower chamber is the inner chamber of the culture plate. Add new cell culture medium in the lower chamber. It is appropriate to just contact the basement membrane of the Transwell cell, which is about 2 ml. Add LGG bacteria diluted with the cell culture medium in the upper chamber, with a final concentration of 10^12^ CFU/ml at 37 ℃, 5% CO_2_, and co culture for 4 h, 12 and 24 h, respectively. Then removed Transwell cell, collected the bacterial suspension, centrifuge at 12,000 R/min for 10 min, discard the supernatant, separate the bacteria, add PBS solution for three times, calculated the final concentration of the bacterial solution.

### Mice experiments design

Male C57BL/6 mice were purchased at 8 weeks of age from the Hunan SJA Laboratory Animal Co., LTD, China. All of the mice were housed at the Animal Laboratory Division, Xiangya School of Public Health, Central South University, China. All animal procedures and testing were conducted according to the National Legislation and local guidelines of the Laboratory Animals Center at the Central South University. The study and research protocols were approved by the Institutional Animal Review Board of Central South University (2020sydw0110). In addition, all animals in the study were treated humanely with regard to the alleviation of suffering. All the mice were maintained in a specific-pathogen-free (SPF) environment with controlled conditions of a 12 h light/dark cycle at 20–22 °C and 45 ± 5% humidity. After 1 week of acclimation, the mice were used for the study with the group design detailed below.

To investigate the effects of LGG or SNHG17 on radiation-induced EMT progress, mice were divided into five groups: control group (Con), radiation-induced lung fibrosis group with 10 Gy radiation treated once time (10 Gy), mice treated with LGG prior to received 10 Gy radiation on lung (10 Gy + LGG), mice treated with siSNHG17 through tail vein injection prior to received 10 Gy radiation on lung (10 Gy + siSNHG17), mice treated with LGG and siSNHG17 prior to received 10 Gy radiation on lung (10 Gy + LGG + siSNHG17). The LGG was administrated for 7 days prior to radiation via water, and the concentration of bacterial suspension was 4.5 × 10^9^ CFU/ml per mouse. Each group was enrolled 10 mice. Mice were subjected to ^60^Co γ-rays irradiation at 10 Gy on the lung part and the other parts of mice were shielded with 10 cm thick lead bricks (dose rate: 200 cGy/min, source-skin-distance: 100 cm, voltage: 180 kV, current: 12.5 mA, the field size: 3 cm × 40 cm) at room temperature at the Institute of Radiation Medicine, Academy of Military Medical Sciences (Beijing, China) [40, 43]. Body weight, food intake and water consumption were recorded. The mice were killed at the 7th weeks to collect tissues for further detection of various parameters.

### RNA isolation and real-time PCR

Total RNA was extracted from cells by using a Total RNA extraction kit (Vazyme, China) according to the manufacturer’s instructions. After the quality and quantity of the extracted RNA were confirmed by a nucleic acid quantitative detector (NanoDrop 2000c, USA), complementary DNA (cDNA) was synthesized using HiScript III RT SuperMix for qPCR (+ gDNA wiper) (Vazyme, China) according to the manufacturer’s instructions. The Taq Pro Universal SYBR qPCR Master Mix (Vazyme, China) was used for real-time PCR analysis on a PCR platform (Bio-Rad CFX96 Touch, USA) to determine the expression level of lncRNA SNHG17. Then, the relative expression of lncRNA SNHG17 was calculated by the 2^−ΔΔCT^ value method, and GAPDH was used as a housekeeping gene. The specific primers for lncRNA SNHG17, PTBP1 and GAPDH used for RT-PCR are listed in Additional file 1: Table S3. All the primers were all synthesized by Sangon (Shanghai, China). Each PCR amplification was performed in triplicate to verify the results. Primers for PCR is listed in Additional file 1: Table S3.

### Western blotting

Cells in the logarithmic growth phase were placed in a 35 mm dish at the appropriate density and cultured in an incubator. Proteins were extracted from irradiated cells by using M-PER Mammalian Protein Extraction Reagent (Thermo Fisher Scientific, Taiwan, China) according to the manufacturer’s instructions. Equal amounts of proteins were separated on a 10% sodium dodecyl sulfate-polyacrylamide gel electrophoresis gel and transferred to nitrocellulose membranes (Millipore, USA). 5% skimmed milk was used to block the membranes for 1 h, and then, the membranes were probed overnight at 4 °C with the primary antibodies listed in Additional file 1: Table S3. Next, the membranes were incubated with specific secondary antibodies (ZSGB-BIO, Beijing, China) for 1 h at room temperature. Peroxidase labeling was visualized via enhanced chemiluminescence labeling using an ECL Western blotting detection system (Thermo Fisher Scientific, Waltham).The details of antibodies used in this study are listed in Additional file 1: Table S4.

### Fluorescence in situ hybridization (FISH)

Cy3-labeled lncRNA SNHG17 probes were designed and synthesized by RiboBio (Guangzhou, China). The probe signals were determined with a Fluorescent In situ Hybridization Kit (RiboBio, Guangzhou, China) following the manufacturer’s guidelines. Images were taken under an immunofluorescence confocal microscope (Crest Optics X-Ligt V3, Italia).

### Immunofluorescence staining

Cells plated on 22 × 22 mm^2^ cover slips in 6-well plates were irradiated and fixed in 4% paraformaldehyde for 30 min at room temperature, permeabilized in 0.25% Triton X-100 buffer for 30 min and then blocked in 3% BSA for 30 min at room temperature. Then, the cells were incubated with a PTBP1 monoclonal antibody (Sino Biological, Beijing, China) and Notch1 antibody (Santa Cruz) antibody at 4 °C overnight and washed twice with PBS. Subsequently, the cells were incubated with a FITC-labeled anti-mouse antibody (Invitroge, USA) and a Texas Red-labeled anti-rabbit antibody (Invitroge, USA) at room temperature for 2 h. The slides were finally fixed with a fluorescent sealer containing DAPI (ZSGB-BIO, Beijing, China). Images were obtained under a confocal microscope (Crest Optics X-Ligt V3, Italia) with the NIS-Elements Viewer 4.20 capture system. We observed 3 high-power visual fields (100× oil lens) were randomly selected from each slice to observe the confocal.

### RNA immunoprecipitation (RIP)

Cells were used to perform RNA immunoprecipitation (RIP) experiments using the Magna RIP™ RNA-Binding Protein Immunoprecipitation Kit (Millipore, Bedford, MA) according to the manufacturer’s instructions. Cells were spread in a 60 mm dish at the appropriate density and were irradiated after they had grown to the appropriate amount. Then, the cells were rinsed with PBS and centrifuged at 1500 rpm for 5 min at 4 °C, and the supernatant was discarded. Next, the cells were re-suspended in 100 µl of RIP lysis buffer and pipetted until homogeneous on ice for 5 min. Magnetic beads were washed with RIP wash buffer and incubated with 5 µg of the anti-IgG antibody (Millipore, Bedford, MA), anti-PTBP1 antibody (Thermo Fisher, USA) for 2 h at room temperature in 100 µl of RIP wash buffer. After a brief centrifugation, the supernatant was discarded, and the unbound protein antibodies on the magnetic beads were washed away with RIP wash buffer. Then, we centrifuged the cell lysate at 4 °C and 12,000 rpm for 10 min and collected 100 µl of the supernatant. The supernatant was incubated with 900 µl of IP buffer containing magnetic beads conjugated with different antibodies at 4 °C overnight. Before incubation, 10 µl of the sample buffer was removed and marked as the input for later Western blot experiments. The sample buffer was then washed with RIP wash buffer and was used in subsequent Western blot experiments for verification after heat denaturation. At the same time, 150 µl of Proteinase K buffer was added to the sample buffer to dissolve protein. Then, immunoprecipitated RNA was isolated, and co-precipitated RNAs were detected by qRT-PCR.

### RNA pulldown assay

The cDNA sequence of SNHG17 and different fragments were cloned into pCDNA3.1 (+). Biotin-labeled RNAs were transcribed in vitro using a biotin-labeling mix and T7 polymerase in the linearized pCDNA3.1 (+) plasmid following the manufacturer’s instructions (Large Scale RNA ProductionSystem-T7, Promega). For the RNA pulldown assay, cells were treated with the RNA 3′-End Desthiobiotinylation Kit and Pierce™ Magnetic RNA-Protein Pull-Down Kit (Thermo Fisher, USA). Cells were rinsed with PBS and then re-suspended in 1 ml ice-cold PBS. Then, we centrifuged the suspension at 4 °C and 1000 rpm for 3 min. Next, the cell pellet was suspended in 400 µl of dilution buffer (with a protease inhibitor cocktail) and centrifuged at 4 °C and 12,000 rpm for 10 min. The cell supernatant was collected for use in the next experiment. Pierce nucleic acid compatible streptavidin magnetic beads were washed twice with wash buffer to remove the stock solution and were re-suspended in RNA capture buffer. We added labeled biotin-lncRNA SNHG17 to the beads and incubated them for 15–30 min. Then, the beads were washed twice with wash buffer and re-suspended in Protein-RNA Binding Buffer. Next, we constructed the RNA pulldown reaction system according to the manufacturer’s instructions and incubated the beads at 4 °C for 2 h in a rotary shaker. Finally, we eluted the proteins with 50 µl biotin elution buffer after washing the beads with wash buffer and detected the proteins by SDS-PAGE and mass spectrometry analysis.

### Cell-cycle analysis

PI/RNase Staining Solution kit (CY2001-P, Sungene Biotech, China) was used. The cells were seeded into 35 mm culture dishes at a density of 70–80% per dish. The cells were transfected or not transfected with siRNA, subjected to irradiation 12 h after the pretreatment, and harvested at the indicated timepoints (0, 2, 4, 6, 8, or 12 h) after irradiation. After the medium was removed, the cells were treated with RNase A (62 µg/ml) and incubated at 37 °C for 30 min. The cells were stained with propidium iodide (PI) solution, and the cell-cycle distribution was analyzed by flow cytometry (Agilent NovoCyte, USA). G2/M assay was based on the cell cycle detection.

### Apoptosis assay

Fluorescein Isothiocyanate (FITC)-Annexin V Apoptosis Detection Kit was used to detect cell apoptosis according to the manufacturer’s instructions (BD Pharmingen, San Diego, CA, USA). Briefly, we collected the cell culture supernatant 24 h after irradiation. After digesting the cells with trypsin (without EDTA), the cells were centrifuged. The supernatant was collected and washed three times with PBS. Then, we re-suspended the cells in binding buffer at a concentration of approximately 5 × 10^5^ cells/ml and added FITC-conjugated Annexin V and propidium iodide (PI) solution according to the manufacturer’s instructions. The cells were incubated at room temperature for 15 min in the dark. Then, the cells were subjected to flow cytometry analysis (Agilent NovoCyte, USA).

### Scratch wound healing migration

Cells were seeded into 6-well plates and grown to approximately 90% confluence. Cell monolayers were scratched with a 20-µl sterile pipette tip. Cells were rinsed with phosphate-buffered saline and cultured in DMEM supplemented with 1% fetal bovine serum. Cell migration was photographed 0 and 48 h after scratching using an inverted microscope (Olympus, Tokyo, Japan).

### Fluorescence in situ hybridization (FISH)

Cy3-labeled lncRNA SNHG17 probes were designed and synthesized by RiboBio (Guangzhou, China). The probe signals were determined with a Fluorescent In situ Hybridization Kit (RiboBio, Guangzhou, China) following the manufacturer’s guidelines. Images were taken under an immunofluorescence confocal microscope (Crest Optics X-Ligt V3, Italia).

### Microarray analysis of lncRNAs

The lncRNA profile between irradiated A549 MLE-12 cells with or without LGG treatment post 6 Gy radiation was performed at oeBiotech Biotechnology Corporation (Shanghai, China). Agilent lncRNA microarray (Agilent Technologies, USA) was used in the analysis. According to the manufacturer’s protocol, lncRNAs were labeled and hybridized with lncRNA complete Labeling and Hybridization kit. Data normalization and processing were performed using Quantile algorithm, Gene Spring Software 12.6 (Agilent Technologies, USA). The differential expression of lncRNAs was performed via the Pearson’s correlation analysis with Cluster 3.0 and TreeView software, and the differentially expressed genes (DEGs) were identified to have at least |logFC| > 2, *p* value < 5% in expression.

### Electron microscopy for structural analysis of the lung

TEM analysis was performed after collection of the lung tissues by Shiyanjia Lab (www.shiyanjia.com). The tissues were split and treated in a cold fixative solution composed of 2.5% glutaraldehyde at 4 °C for 4 h. After washing with PBS, the specimens were post-fixed in 1% OsO4 at 4 °C for 1 h and washed again with PBS. A graded series of ethanol solutions was used for further dehydration, and the specimens were transferred to be incubated. TEM was performed with a JEM-2100 F at 80 kV, and images were acquired using a side-inserted BioScan camera.

### Online available databases

SNHG expression in various cancers was evaluated using the TCGA database (https://cistrome.shinyapps.io/timer/). The GEPIA database (http://gepia.cancer-pku.cn/index. html) was used to analyze RNA sequencing data from normal and tumor tissue samples from the TCGA and GTEx projects. We also used the GEPIA website to generate overall free survival rates. UCSC (http://genome.ucsc.edu/) and ALGGEN (http://alggen.lsi.upc.es/) databases were used to obtain the potential transcriptional binding sites in the promoter of genes. GeneMainia (http://genemania.org/) and Hitpredict (http://www.hitpredict.org/) databases was used to predict the potential interaction proteins with SNHG17.

### Statistical analysis

All experiments were performed with at least three independent experiments. In general, Student’s two-tailed unpaired t test was used to compare differences between two groups. One-way analysis of variance followed by the Newman–Keuls multiple comparison test were used to compare more than two groups. All data are expressed as the means ± standard deviation (SD) for each experiment. A *p* value of < 0.05 was considered to indicate a statistically significant result. GraphPad Prism 6 Software (GraphPad Software Inc., La Jolla, CA) was utilized for all statistical analyses and construction of graphs.

## Supplementary Information

**Additional file 1**. Supplementary Figures and Tables.

## Data Availability

Supporting data required based on the corresponding author.
