# Supplementary material for: Lactobacillus rhamnosus GG ameliorates radiation-induced lung fibrosis via lncRNASNHG17/PTBP1/NICD axis modulation
Source: Biol Direct. 2023 Jan 12;18:2. doi: 10.1186/s13062-023-00357-x (PMC9835385; doi:10.1186/s13062-023-00357-x)
Supplement: Supplementary file 1 — Additional file 1. Supplementary Figures and Tables. [file 13062_2023_357_MOESM1_ESM.docx]

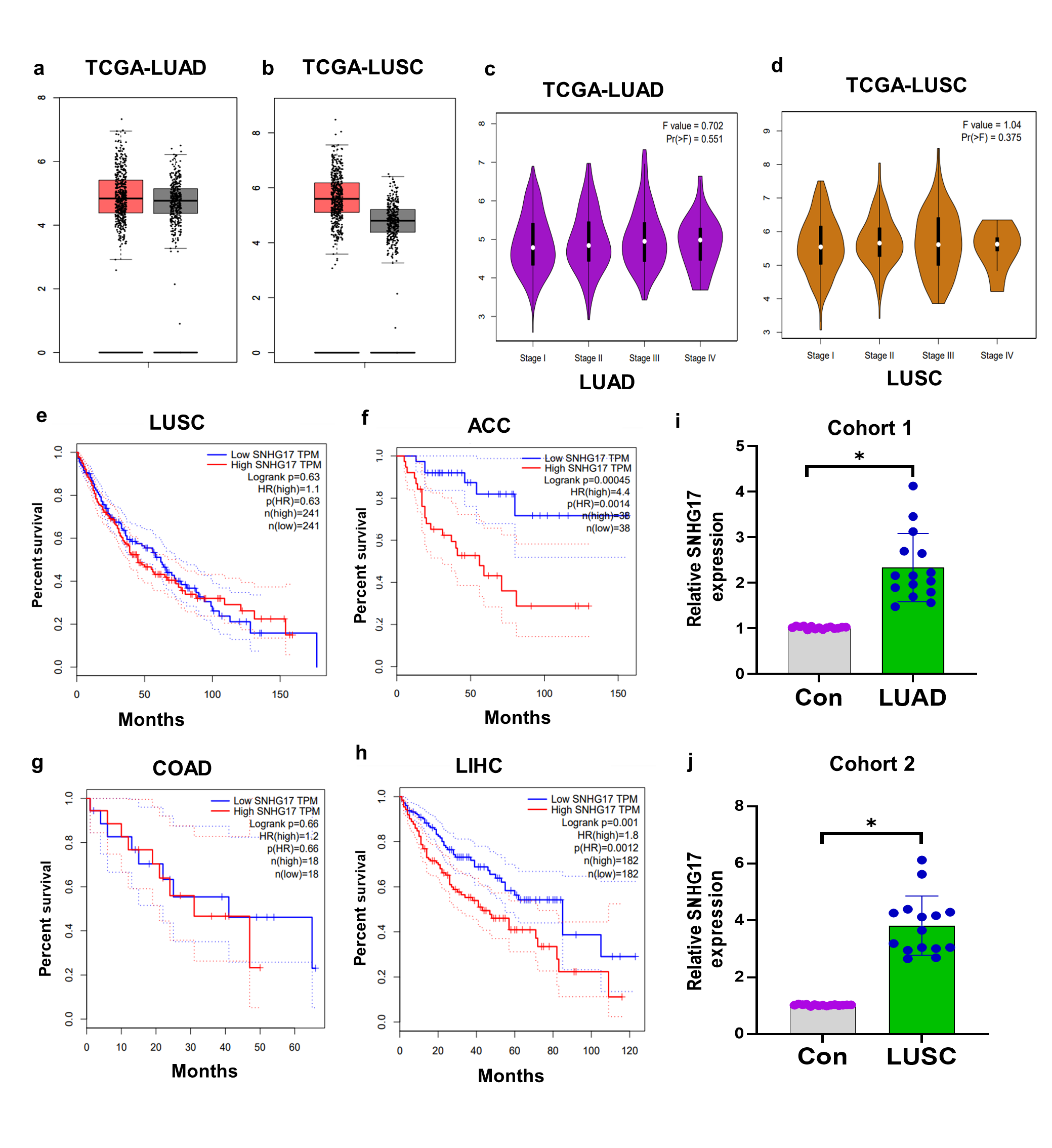


Supplementary Figure 1. The expression of SNHG17 in various tissues. a. Comparison analysis of expression of SNHG17 in lung adenocarcinoma (LUAD) tissues with adjacent normal tissues in online database of GEPIA (http://gepia.cancer-pku.cn/index.html). b. Comparison analysis of expression of SNHG17 in lung squamous cell carcinoma (LUSC) tissues with adjacent normal tissues in online database of GEPIA(<http://gepia.cancer-pku.cn/index.html>). c. Comparison analysis of expression of SNHG17 in lung adenocarcinoma (LUAD) tissues in various stage (<http://gepia.cancer-pku.cn/index.html>). d. Comparison analysis of expression of SNHG17 in lung squamous cell carcinoma (LUSC) tissues in various stage (<http://gepia.cancer-pku.cn/index.html>). e. Over survival analysis of high or low levels of SNHG17 in LUSC cancer patients in online database of GEPIA(<http://gepia.cancer-pku.cn/index.html>). f. Over survival analysis of high or low levels of SNHG17 in Adrenocortical carcinoma(ACC) cancer patients in online database of GEPIA(<http://gepia.cancer-pku.cn/index.html>). g. Over survival analysis of high or low levels of SNHG17 in Colon adenocarcinoma(COAD) cancer patients in online database of GEPIA(<http://gepia.cancer-pku.cn/index.html>). h. Over survival analysis of high or low levels of SNHG17 in Liver hepatocellular carcinoma (LIHC) cancer patients in online database of GEPIA (<http://gepia.cancer-pku.cn/index.html>). i. The expression of SNHG17was measured in 15 LUAD tissues and 15 non-cancerous tissue samples by RT-qPCR. β-actin was used as an internal reference. j. The expression of SNHG17was measured in 15 LUSC tissues and 15 non-cancerous tissue samples by RT-qPCR. β-actin was used as an internal reference. Data are means ±SD (standard deviation). N = 3 independent experiments, Student’s two-tailed unpaired t test was used to compare differences between two groups. *p < 0.05. Supplementary Figure 2. SNHG17 expression is associated with IR in a dose- and time-dependent manner. a. Quantitative analysis of SNHG17 expression in HBE, BEAS2B, A549 and H1299 cells by qRT-PCR. b. Quantitative analysis of SNHG17 expression in A549 cells at 24 h post indicated dosage radiation by qRT-PCR. c. Quantitative analysis of SNHG17 expression in A549 cells at indicated timpoints post 6Gy by qRT-PCR. d. Quantitative analysis of SNHG17 expression in A549 cells treated with LGG with indicated MOIs with 6 h. e. Quantitative analysis of SNHG17 expression in A549 cells treated with LGG or dead LGG for 24 h at indicated MOIs. f. Representative images of A549 cells at 24h following treated with or without LGG or radiation. Scale bar=50μm. g. Quantitative analysis of SNHG17 expression in A549 cells treated with or without LGG post radiation. h. Representative images of the RNA FISH and IF assay to determine the subcellular localization of SNHG17 in A549 cells treated with or without LGG post radiation. Data are means ±SD (standard deviation). N = 3 independent experiments, Student’s two-tailed unpaired t test was used to compare differences between two groups. *p < 0.05.


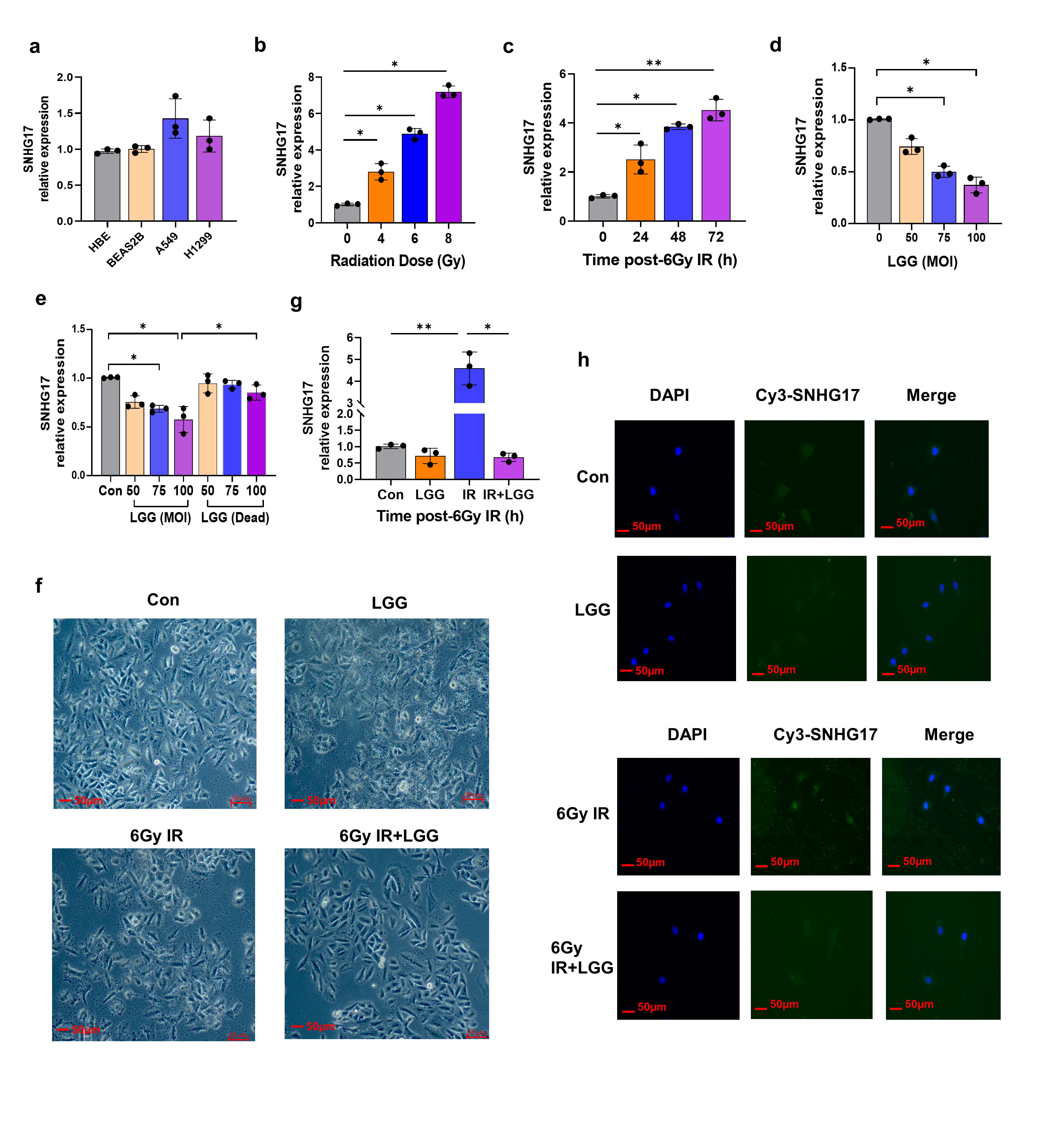

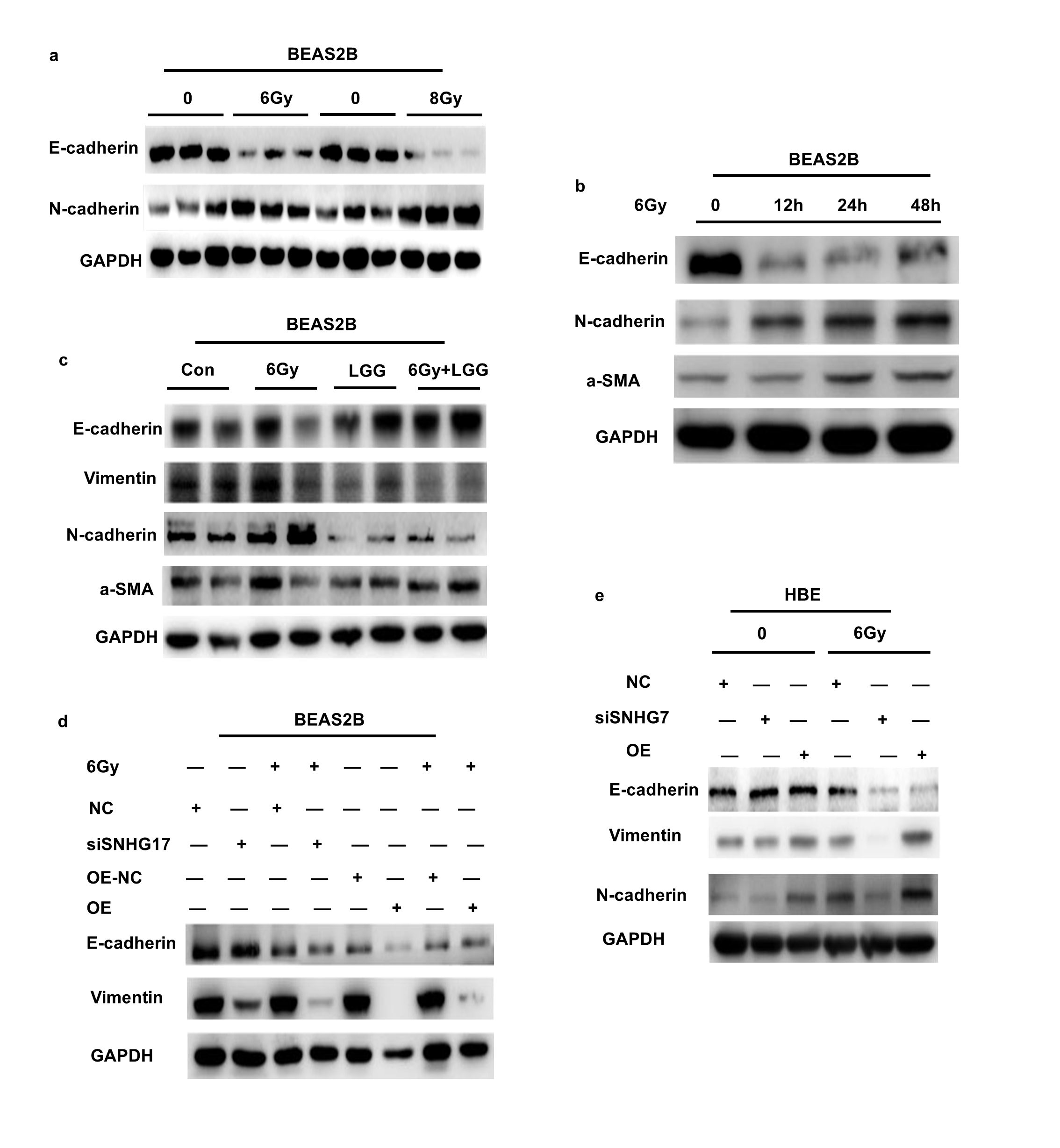


Supplementary Figure 3. SNHG17 is associated with the regulation of EMT process. a. Representative blots E-cadherin, and N-cadherin protein expression in lung tissues with or without radiation in BEAS2B cells by Western blotting assay. b. Representative blots E-cadherin, and N-cadherin and a-SMA protein expression in BEAS2B cells at indicated timepoints post radiation by Western blotting assay. c. Representative blots E-cadherin, and N-cadherin and Vimentin protein expression in BEAS2B cells with or without LGG treatment post radiation by Western blotting assay. d. Representative blots E-cadherin, and Vimentin protein expression in BEAS2B cells with knockdown or overexpression of SNHG17 expression post radiation by Western blotting assay. e. Representative blots Vimentin and N-cadherin protein expression in HBE cells with knockdown of or overexpression of SNHG17 expression post radiation by Western blotting assay.

Supplementary Table 1 Top 20 differential expressed lncRNAs in the LGG +IR vs. IR A549 cells post 6Gy radiation.

| **Upregulated lncRNA** | | | |  | **Downregulated lncRNA** | | | |
| --- | --- | --- | --- | --- | --- | --- | --- | --- |
| **Gene-ID** | **Fold change** | **Log2**  **(Fold)** | ***p* value** |  | **Gene-ID** | **Fold change** | **Log2**  **(Fold)** | ***p* value** |
| ENST00000609564 | 95.428 | 6.576341 | 0.001242 |  | lncRNA SNHG17 | 0.016987 | -5.87945 | 0.011763 |
| ENST00000610988 | 83.35608 | 6.381216 | 7.16E-05 |  | NR_111954.1 | 0.018008 | -5.79521 | 0.007021 |
| ENST00000518481 | 61.5625 | 5.94398 | 0.015315 |  | XR_943833.2 | 0.020736 | -5.59173 | 0.010488 |
| ENST00000435966 | 48.86889 | 5.610845 | 0.020639 |  | ENST00000651474 | 0.02334 | -5.42107 | 0.040276 |
| NR_134567.1 | 46.48616 | 5.538729 | 0.001856 |  | ENST00000431616 | 0.024436 | -5.35483 | 0.003461 |
| ENST00000505709 | 33.55813 | 5.068591 | 0.007759 |  | NR_110130.1 | 0.026932 | -5.21454 | 0.017899 |
| ENST00000437331 | 33.5568 | 5.068533 | 0.020327 |  | XR_002959406.1 | 0.027819 | -5.16776 | 0.043448 |
| ENST00000632919 | 33.18902 | 5.052634 | 0.034073 |  | ENST00000642205 | 0.031924 | -4.96919 | 0.03722 |
| XR_923256.3 | 28.65114 | 4.84052 | 0.011771 |  | TCONS_00000658 | 0.032191 | -4.95718 | 0.016136 |
| ENST00000531092 | 25.07219 | 4.648016 | 0.00257 |  | NR_135062.1 | 0.032368 | -4.94928 | 0.042086 |
| XR_001743946.1 | 23.23765 | 4.538392 | 0.039154 |  | ENST00000606533 | 0.034656 | -4.85076 | 0.031977 |
| TCONS_00016003 | 19.35036 | 4.274289 | 0.008377 |  | ENST00000618708 | 0.037696 | -4.72943 | 2.54E-05 |
| ENST00000456651 | 19.00272 | 4.248134 | 0.037854 |  | NR_110176.1 | 0.040367 | -4.63067 | 2.27E-08 |
| NR_110295.2 | 18.86414 | 4.237574 | 0.017299 |  | XR_001754617.2 | 0.042842 | -4.54484 | 0.018351 |
| NR_148975.1 | 18.16168 | 4.182826 | 0.007432 |  | XR_937715.3 | 0.04571 | -4.45136 | 0.003546 |
| ENST00000587078 | 18.01367 | 4.17102 | 0.018816 |  | XR_001740863.2 | 0.049896 | -4.32494 | 0.016459 |
| ENST00000652496 | 16.75296 | 4.066344 | 0.029671 |  | XR_001755046.2 | 0.050441 | -4.30926 | 0.022034 |
| NR_021490.2 | 16.7263 | 4.064046 | 0.005019 |  | ENST00000639159 | 0.063168 | -3.98466 | 0.03257 |
| ENST00000634265 | 15.45594 | 3.95009 | 0.040577 |  | XR_948174.2 | 0.064568 | -3.95305 | 0.007872 |
| NR_146383.1 | 15.35297 | 3.940446 | 0.025886 |  | ENST00000577938 | 0.065163 | -3.93981 | 0.032066 |

*P*<0.05 represents significantly changed.

Supplementary Table 2 Top 20 potential binding proteins in the siSNHG17+6GyIR vs. siSNHG17 in A549 cells by LC-MS/MS analysis.

| **Upregulated proteins** | | | |  | **Downregulated proteins** | | | |
| --- | --- | --- | --- | --- | --- | --- | --- | --- |
| **Gene-ID** | **Fold change** | **Log2**  **(Fold)** |  |  | **Gene-ID** | **Fold change** | **Log2**  **(Fold)** |  |
| UBA52 | 200 | 7.643856 |  |  | PTBP1 | 0.144767 | -2.7882 |  |
| CSTB | 200 | 7.643856 |  |  | MYH10 | 0.160626 | -2.63822 |  |
| RPS14 | 200 | 7.643856 |  |  | MYH14 | 0.327026 | -1.61252 |  |
| PKP1 | 200 | 7.643856 |  |  | MYO1D | 0.328652 | -1.60537 |  |
| HSPB1 | 200 | 7.643856 |  |  | MYH9 | 0.41127 | -1.28184 |  |
| SERPINB12 | 200 | 7.643856 |  |  | MATR3 | 0.42441 | -1.23647 |  |
| ANXA1 | 200 | 7.643856 |  |  | ACTB | 0.485546 | -1.04232 |  |
| HBA2 | 200 | 7.643856 |  |  | GSN | 0.530462 | -0.91468 |  |
| DEFA3 | 200 | 7.643856 |  |  | RPL23 | 0.562276 | -0.83065 |  |
| HSP90AB1 | 200 | 7.643856 |  |  | ACTA1 | 0.565048 | -0.82355 |  |
| IGHV3OR15-7 | 200 | 7.643856 |  |  | MYL6 | 0.611875 | -0.70869 |  |
| LDHA | 200 | 7.643856 |  |  | RPS26 | 0.62719 | -0.67303 |  |
| XP32 | 200 | 7.643856 |  |  |  |  |  |  |
| MYO1B | 200 | 7.643856 |  |  |  |  |  |  |
| RPS3 | 200 | 7.643856 |  |  |  |  |  |  |
| ALB | 200 | 7.643856 |  |  |  |  |  |  |
| GAPDH | 200 | 7.643856 |  |  |  |  |  |  |
| RPS16 | 200 | 7.643856 |  |  |  |  |  |  |
| ENSG00000231767 | 200 | 7.643856 |  |  |  |  |  |  |
| RAN | 200 | 7.643856 |  |  |  |  |  |  |

Supplementary Table 3 Primary primers used in this study

| siRNA/shRNA/probe name | sequence |
| --- | --- |
| Negative control sense | 5' TTCTCCGAACGTGTCACGTdTdT 3' |
| Negative control antisense | 5' ACGTGACACGTTCGGAGAAdTdT3' |
| lncRNA-SNHG17 shRNA-1 sense | 5' GGTGACGTGTCTTCAAGAATT 3' |
| lncRNA-SNHG17 shRNA-1 antisense | 5' TTCTTGAAGACACGTCACCTT 3' |
| lncRNA-SNHG17 shRNA-2 sense | 5' CCTGGAATGACTTAATAATT 3' |
| lncRNA-SNHG17 shRNA-2 antisense | 5' TTATTAAAGTCATTCCAGGTT 3' |
| PTBP1 shRNA-1 sense | 5' CCCTCATTGACCTGCACAATT 3' |
| PTBP1 shRNA-1 antisense | 5' TTGTGCAGGTCAATGAGGGTT 3' |
| lncRNA-SNHG17 Forward | 5' TGCGTGGGTAAAGTCTCAGC 3' |
| lncRNA-SNHG17 Reverse | 5' TGACGCTTCATGTGGTAGCC 3' |
| PTBP1 forward | 5' CTCACCAGCCTCAACGTCAA 3' |
| PTBP1 Reverse | 5' TTATACCAGGTGCACCGAAGG 3' |
| GAPDH Forward | 5' GGTGGTCTCCTCTGACTTCAACA 3' |
| GAPDH Reverse | 5' GTTGCTGTAGCCAAATTCGTTGT 3' |

| Antibody | Application | Company | Catalog numbers |
| --- | --- | --- | --- |
| PTBP1 | 1:500 for IF | Sino Biological | 101043-T46 |
| PTBP1 | 1:1000 for WB, 1:1000 for IP and RIP | Thermo Fisher | #32-4800 |
| Notch1 | 1:1000 for WB | Santa Cruz | sc-373891 |
| E-cadherin | 1:1000 for WB | Cell Signaling Technology | #14472 |
| N-cadherin | 1:1000 for WB | Cell Signaling Technology | #13116 |
| Vimentin | 1:1000 for WB | Cell Signaling Technology | #5741 |
| β-actin | 1:1000 for WB | ZS-bio | TA-09 |
| GAPDH | 1:1000 for WB | ZS-bio | TA-08 |

Supplementary Table 4 Primary antibodies used in this study
